# Synchronous Renal Cell Carcinoma and Gastrointestinal Malignancies

**DOI:** 10.1155/2016/7329463

**Published:** 2016-01-19

**Authors:** Tamer J. Dafashy, Cameron K. Ghaffary, Kyle T. Keyes, Joseph Sonstein

**Affiliations:** Department of Surgery, Division of Urology, University of Texas Medical Branch, 301 University Boulevard, Galveston, TX 77555, USA

## Abstract

While renal cell carcinoma is the most commonly diagnosed neoplasm of the kidney, its simultaneous diagnosis with a gastrointestinal malignancy is a rare, but well reported phenomenon. This discussion focuses on three independent cases in which each patient was diagnosed with renal cell carcinoma and a unique synchronous gastrointestinal malignancy. Case 1 explores the diagnosis and surgical intervention of a 66-year-old male patient synchronously diagnosed with clear cell renal cell carcinoma and a carcinoid tumor of the small bowel. Case 2 describes the diagnosis and surgical intervention of a 61-year-old male found to have clear cell renal cell carcinoma and a mucinous appendiceal neoplasm. Lastly, Case 3 focuses on the interventions and management of a 36-year-old female diagnosed with synchronous clear cell renal carcinoma and hereditary nonpolyposis colorectal cancer. This case series examines each distinct patient's presentation, discusses the diagnosis, and compares and contrasts the findings while discussing the literature on this topic.

## 1. Introduction

Renal cell carcinoma (RCC) is the most common neoplasm of the kidney, affecting 15.5 people per 100,000 men and women each year and accounting for only 3.8% of new cancer cases diagnosed annually in the United States [1]. While much more uncommon, the number of new cases of small bowel malignancies accounts for 2.1 per 100,000 men and women, with an overall incidence less than 1% of all primary cancers. Colorectal cancer accounts for the majority of diagnosed gastrointestinal cancers, with an incidence of 43.7 people per 100,000 men and women each year and accounting for 8.2% of total cancer cases diagnosed each year [1, 2]. Although the occurrence of each malignancy is relatively low, the incidence of RCC and gastrointestinal malignancy, whether small bowel or colorectal, occurring simultaneously is undoubtedly rare [3–7]. The purpose of this case series is to describe three unique patients who presented with synchronous RCC and distinct gastrointestinal neoplasms and to discuss the literature on this topic.

This discussion utilizes the Warren and Gates definition to identify synchronous tumors: each tumor must present with a definite picture of malignancy, each must be distinct, and the possibility that one is a metastasis of the other must be excluded [8]. The etiology and pathogenesis of synchronous tumors have yet to be fully understood, although some hypothesize that concurrent tumors can arise from tissues with similar embryological origins. This may occur when these tissues are simultaneously affected by factors like carcinogens, such as tobacco and alcohol; hormones; genetic alterations; chemotherapy treatment; or radiotherapy [7].

It has been well reported that RCC is associated with other primary malignancies, including prostate, bladder, and rectal cancers, as well as non-Hodgkin's lymphoma [9]. We report three distinct cases of synchronous RCC and gastrointestinal cancer treated in our institution, specifically synchronous RCC and carcinoid tumor (Case 1), RCC and appendiceal mucinous neoplasm (Case 2), and RCC and hereditary nonpolyposis colorectal cancer (Case 3).

## 2. Case Presentations

### 2.1. Case 1: RCC + Carcinoid Tumor

A 66-year-old man presented to the clinic with a history of acute onset right lower quadrant abdominal pain, hematuria, and flank pain. General physical examination revealed no significant findings. The patient's family history revealed his mother had both thyroid and colon cancer, though the details of each were unknown.

Initial laboratory findings included mild iron deficiency anemia with elevated white blood cell count, increased BUN and creatinine, and a urine analysis positive for blood and protein. Abdominal CT scan revealed a hypervascular multilocular solid renal mass that had compromised Gerota's fascia and was occupying the right renal vein. Also noted on CT scan was an enhancing partially necrotic solid nodule within the left mesentery, as well as multiple diminutive ill-defined hepatic lesions.

The patient then underwent a right radical nephrectomy with tumor thrombectomy and retroperitoneal lymph node dissection. During intraoperative inspection of the mesenteric region, an ileal mass was encountered, and an* en bloc* resection was performed with primary anastomosis and ultrasound-guided liver biopsies.

**Figure 1:**
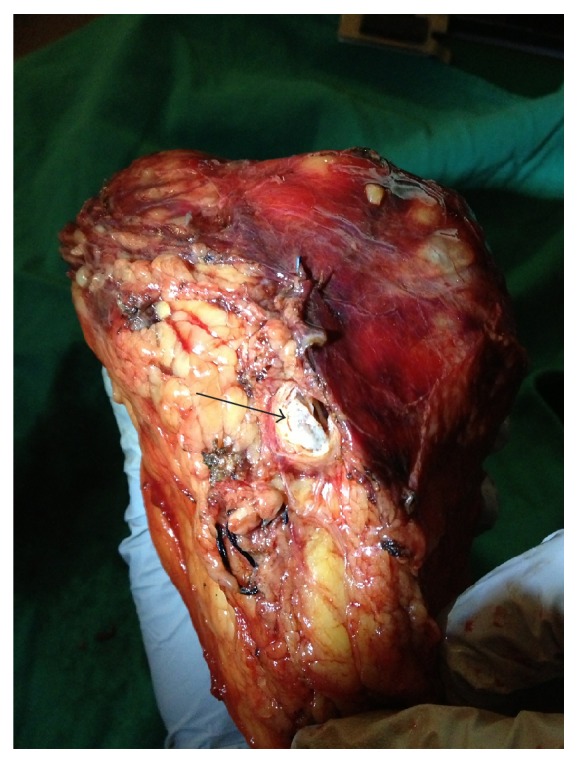
The surgically resected specimen consisted of clear cell renal carcinoma with tumor thrombus (arrow).

**Figure 2:**
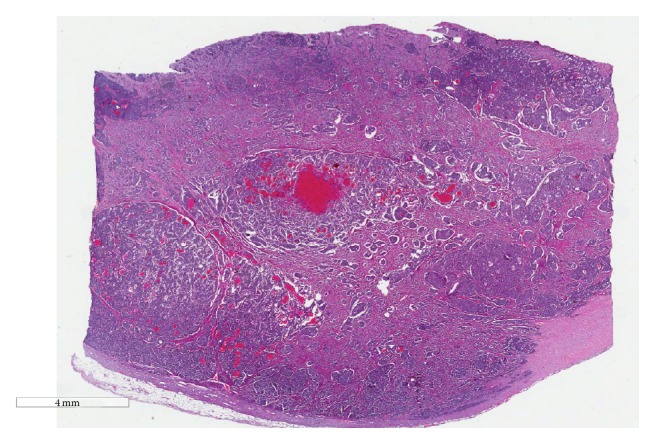
Histological examination (hematoxylin-eosin staining; magnification 4x) of the resected small bowel specimen showing a neuroendocrine carcinoma composed of islands of uniform cells embedded within fibrous tissue.

Evaluation of the nephrectomy specimen revealed a 7 cm Fuhrman grade 2 clear cell RCC invading the renal vein with lymphovascular invasion and clear surgical margins without invasion of Gerota's fascia or the renal sinus (T3aN0M0) (Figure 1). The resected small bowel specimen demonstrated a 2.5 cm ileal mass with multiple foci of intermediate grade neuroendocrine carcinoma (G2 with 12 mitoses per high-power field) seen in Figure 2 penetrating the serosal surface of the bowel with both lymphovascular and perineural invasion. Interestingly 2/15 mesenteric lymph nodes, 8/13 paracaval nodes, and 3 mesenteric nodules were positive for neuroendocrine carcinoma (T4N1M0). Liver biopsy revealed the presence of localized hemangiomas. Following successful resection of both tumors, his brain MRI; CT of the chest, abdomen, and pelvis; and octreotide scan found no evidence of additional metastatic disease. Preoperative 24-hour urine for 5-HIAA was mildly elevated but postoperative repeat was normal. The patient recovered well after surgery and continues to follow up regularly with no signs of reoccurrence in 20 months after surgery.

### 2.2. Case 2: RCC + Mucinous Appendiceal Neoplasm

**Figure 3:**
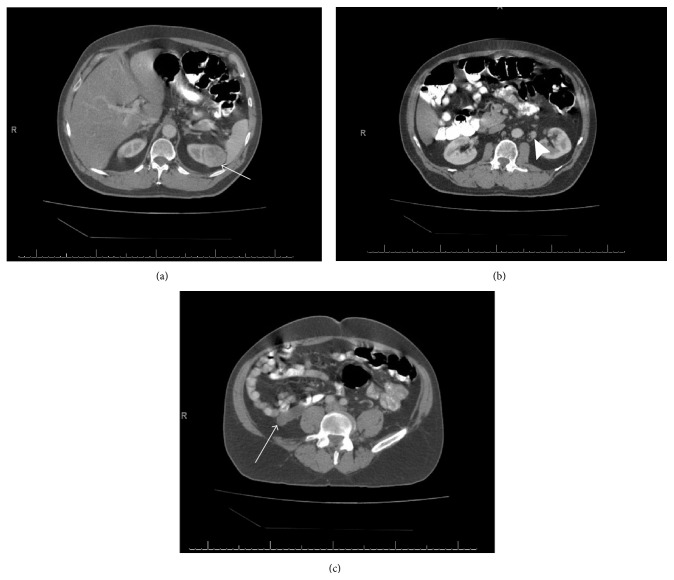
CT scans reveal a partially enhancing mass ((a) arrow) located on the upper pole of the left kidney. The presence of para-aortic lymphadenopathy ((b) arrowhead) as well as a prominent appendiceal mass ((c) arrow) was also noted.

A 61-year-old man presented with constant dull right lower quadrant pain associated with fatigue and malaise. Initial CT scan showed an enlarged appendix and a partially exophytic, enhancing mass located in the upper pole of the left kidney with an enlarged para-aortic lymph node (Figure 3). The patient then underwent appendectomy; pathology findings showed a low-grade appendiceal mucinous neoplasm. After recovery, the patient was then referred to the urology service for assessment and treatment of the left renal mass.

Initial examination of the patient revealed an asymptomatic well-developed man. Besides a past medical history of hypertension, the patient was otherwise healthy. Upon physical examination, findings included bilaterally descended testes, left grade II varicocele, and lack of lymphadenopathy or hernia. The patient's family history was insignificant. The patient's CBC and serum chemistry panel were normal. Urinary analysis found 3 RBCs per high-power field but was otherwise normal.

The patient then underwent left-sided robotic-assisted partial nephrectomy with para-aortic lymphadenectomy. Histological findings of the renal mass confirmed a 3 cm Fuhrman grade 3, clear cell RCC limited to the renal parenchyma with negative margins. Resection of lymph nodes revealed 0/6 perihilar and 1/14 para-aortic nodes with metastasis. He was, therefore, staged as a T1aN1M0. Following removal of tumors, the patient recovered without complication. A follow-up CT scan was performed four months postoperatively and showed no evidence of metastasis. The patient is currently in good health and has no evidence of disease at four-month follow-up.

### 2.3. Case 3: RCC + Hereditary Nonpolyposis Colorectal Cancer

A 36-year-old female initially presented for evaluation of a left supraclavicular neck mass. The patient underwent fine needle aspiration of the mass, which revealed adenocarcinoma favoring renal cell. CT scan of the chest, abdomen, and pelvis revealed a large left renal mass 9 cm in diameter. The scan incidentally also detected a possible filling defect in the transverse colon. The patient underwent colonoscopy, and a mass was visualized and biopsied. Histological examination found the mass to be positive for invasive adenocarcinoma.

Physical examination of the patient revealed an overweight woman with a past medical history of hypertension. The patient denied any changes in bowel habits or urinary complaints other than intermittent left flank pain. The patient reported that her family history was negative for cancer or polyps. General physical examination revealed a small nodule palpable in the left supraclavicular region of the patient's neck. Laboratory findings were normal except for presence of iron deficiency anemia.

**Figure 4:**
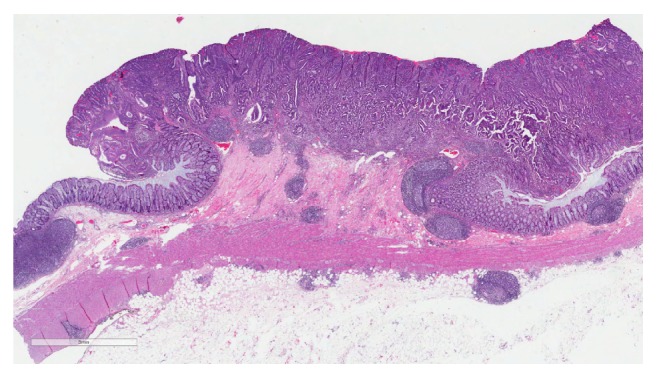
Histological examination (hematoxylin-eosin staining; magnification 3x) of the resected large bowel specimen showing a moderately differentiated adenocarcinoma invading into the submucosa.

The patient then underwent left radical nephrectomy with periaortic lymphadenectomy and extended right hemicolectomy. Histological examination of the left kidney confirmed a 7.5 cm Fuhrman grade 4, clear cell RCC limited within Gerota's fascia with no lymphovascular invasion. The tumor was present in 5/5 periaortic lymph nodes that were resected (T2aN1Mx). The resected large bowel specimens demonstrated tubular adenoma present in the cecum and hepatic flexure; also noted was moderately differentiated invasive adenocarcinoma extending into the submucosa of the transverse colon (T1N0M0) (Figure 4). Microsatellite instability staining was ordered; results were positive for hereditary nonpolyposis colorectal cancer (HNPCC). Given this patient's negative family history of colon cancer, this was likely a spontaneous mutation. The patient developed chylous ascites and peritonitis with acute kidney injury postoperatively and returned to the operating room for evacuation of fluid and ileostomy placement. She eventually recovered following a cardiac diet (low fat, low cholesterol, and high protein) with peritoneal drainage and octreotide.

She was started on sunitinib postoperatively but failed therapy after developing bilateral malignant pleural effusion and malignant disease progression and was subsequently given temsirolimus.

After one month, postoperative CT scan revealed local recurrence in the kidney bed, suggesting highly aggressive cancer. Imaging also found enhancing enlarged supraclavicular lymph nodes extending cranially along the jugular chain and caudally into the mediastinum. This finding was strongly suggestive of metastatic disease. PET scan was performed and confirmed diffuse nodal metastasis throughout the thorax and neck. She then underwent chest wall biopsy and pleurodesis using video-assisted thoracoscopic surgery, the results of which confirmed malignant RCC. The patient died approximately one year following initial resection.

## 3. Discussion

The occurrence of synchronous malignancies has been well established in the literature. In the case of RCC, multiple primary malignancies associated with this neoplasm have been the focus of many studies within the past decade. Beisland et al. [3] used the national Cancer Registry of Norway to determine the rates association of RCC in conjunction with other cancers. They found the rate of multiple primary malignancies associated with RCC was 16.1%, supporting previous studies that reported prostate, breast, colorectal, bladder, non-Hodgkin's lymphoma, and lung cancer to be the most common primary cancers in patients with RCC. Specifically they reported 3.7% of those diagnosed with RCC had developed a synchronous tumor. In the case of synchronous colorectal and RCC cancers, the incidence is widely varied. Capra et al. [4] estimate 0.03–0.5% and O'Boyle and Kemeny [6] report an incidence of 0.5%, while Halak et al. [5] describe 4.85%, though they admit this value probably does not reflect the true incidence. Regardless of actual incidence, it is clear that RCC is associated with a higher incidence of multiple primary malignancies.

The etiology and pathogenesis of multiple primary malignancies have yet to be explained. It has been thought that interplay of genetic and environmental risk factors common to both cancers could cause multiple malignancies to arise. Common risk factors include tobacco, pollution, ultraviolet light, therapeutic chemotherapy and radiotherapy, and endocrine factors. It is thought that these factors may act individually or in combination [10]. Interestingly however, a previous study reported synchronous renal and colon malignancies unrelated to hereditary factors [6].

Considering the three cases presented, each patient did not smoke and had no prior medical illness that could point to a common risk factor. The patient presented in Case 1 reported a family history of thyroid and colon cancer. A possible underlying genetic variation may have been present, although no genetic testing was performed on either the first or the second patient presented. The third case discussed demonstrates a well-established genetic predisposing factor. Individuals with HNPCC are known to have various mismatch repair genes that are functionally affected, with patients having 80% lifetime risk of developing colon cancer [11], as well as an increased risk of developing extracolonic tumors, including endometrial, ovarian, ureteral, and renal cancers [12]. These latter patients who also develop extracolonic manifestations in addition to colorectal cancer, such as the patient in Case 3, are a specific subset of HNPCC patients referred to as having Lynch syndrome II. These patients are distinct from those with Lynch syndrome I, whose disposition to develop malignancies is limited to the colon. Differentiating between the two syndromes is often difficult as there is no difference with respect to sex, age, occurrence, progression, or the site distribution of cancer in the colon. Both Lynch syndromes I and II are characterized by an autosomal dominant tendency to develop multiple primary colonic malignancies early in life, with the distinction separating the subtypes relying on the presence or lack of extracolonic features [13]. Given this knowledge, Papalampros et al. [7] suggest microsatellite instability testing could be applied to all patients with colorectal and urological cancers to assist in detecting a common genetic aberration between malignancies. Further studies with this focus may be an initial building block in the bridge between RCC and the demonstrated association with primary colorectal malignancies.

A common finding among the three clinical cases presented was that, in each case, the renal malignancy was aggressive clear cell RCC. This may be a coincidental finding, as clear cell is the most common form of RCC [14]. Others have reported that patients with either papillary RCC or chromophobe RCC are more likely to have secondary colon cancer compared to patients with clear cell RCC; however, the literature lacks information regarding the histological subtype of RCC and synchronous gastrointestinal tumors [15]. Another common finding among the three cases was that the RCC was incidentally found on CT scan. This reflects the wide use of diagnostic imaging as part of the standard of care utilized when evaluating patients. Through this report, we acknowledge and affirm the findings of Halak et al. [5], who described the use of preoperative imaging studies as an invaluable tool for detecting synchronous asymptomatic renal lesions in patients with colorectal cancer.

Current guidelines by the American Urological Association regarding the management of clinical stage 1 renal masses lack an explicit recommendation for clinicians to be aware of the possibility of a secondary primary malignancy [16]. However, based on this clinical case series and the literature considered, the presence of RCC with a synchronous primary neoplasm is a substantial clinical entity that should be acknowledged upon evaluation of such patients. Specifically in patients with RCC, we believe practicing physicians should have a heightened awareness of the risk of a simultaneous separate primary malignancy and approach patients' new concerns or symptoms with diligence.
